# Prognostic significance of long non-coding RNA five prime to XIST in various cancers

**DOI:** 10.1186/s12885-021-09161-0

**Published:** 2022-01-13

**Authors:** Jian Zhou, Junjie Chen, Ziyuan Chen, Gen Wu, Zhen Zhou, Tong Wu, Wanchun Wang, Yingquan Luo, Tang Liu

**Affiliations:** 1 Department of Orthopedics, The Second Xiangya Hospital of Central South University, Changsha, Hunan 410011 China; 2 Department of Orthopedics, Longhui People’s Hospital, Shaoyang, Hunan 422200 China; 3 Department of Orthopedics, The First People’s Hospital of Changde City, Changde, Hunan 415003 China; 4 Menzies Institute for Medical Research, University of Tasmania, Hobart, Tasmania Australia; 5 Department of Emergency, The First Hospital of Changsha City, Changsha, Hunan 410005 China; 6 Department of General Medicine, The Second Xiangya Hospital of Central South University, Changsha, Hunan 410011 China

**Keywords:** LncRNA FTX, Clinicopathological features, Prognosis, Meta-analysis, TCGA dataset

## Abstract

**Background:**

To observe the clinicopathological and prognostic value of long non-coding RNA five prime to X inactive specific transcript (lncFTX) in multiple tumors.

**Methods:**

Eligible studies for lncFTX were identified by searching PubMed, Embase, Web of Science and Cochrane Library databases from inception to December 01, 2020. Stata 12.0 software was used to calculate the odds ratio (OR)/hazard ratio (HR) and 95% confidence interval (95% CI). We used The Cancer Genome Atlas (TCGA) dataset to further investigate the differential expression and prognostic value of lncFTX.

**Results:**

We included 11 studies involving a total of 1633 patients. The results showed that the expression of lncFTX was positively associated with advanced TNM stage (III-IV versus I-II) (OR = 2.30, 95% CI: 1.74–3.03, *P* < 0.05), lymph nodes metastasis (OR = 3.01, 95% CI: 2.00–4.52, P < 0.05), distant metastasis (OR = 3.68, 95% CI: 2.13–6.34, P < 0.05), and cancer mortality (HR = 1.83, 95% CI: 1.20–2.81, P < 0.05). However, the expression of lncFTX was not associated with tumor differentiation (poor differentiation versus well or moderate differentiation) and vessel invasion of cancer. Subgroup analysis showed that the higher lncFTX expression was associated with shorter overall survival in cancer patients, regardless of the sample size and cancer type. No publication bias was found, and the sensitivity analysis results suggested that the main findings were robust. Elevated expression and prognostic significance of FTX were confirmed using TCGA dataset.

**Conclusions:**

This study found that the expression of lncFTX was positively associated with advanced tumor node metastasis (TNM) stage, lymph nodes, distant metastasis and, cancer mortality, suggesting that lncFTX might be a potential prognostic biomarker for tumors.

**Supplementary Information:**

The online version contains supplementary material available at 10.1186/s12885-021-09161-0.

## Introduction

Cancer is a leading cause of death worldwide and an important public health problem. In the United States, 606,880 cancer related deaths and 1,762,450 new cancer cases were projected to occur in 2019 [1]. Although substantial advances in cancer treatments have been made in recent years, patients’ prognosis in response to these therapies remains unsatisfactory. The five-year survival rates of patients with most cancer types are relatively low. One of the main reasons that lead to the poor prognosis in cancer patients is that the failure to detect the cancer at early stage. Most cancer patients are already at the advanced or metastatic stage at the time of diagnosis. The delay in cancer diagnosis will lead to a delay in cancer treatment, and further result in stage progression, disease worsening and eventually death [2, 3] Therefore, there is an urgent need to find biomarkers that can help clinicians diagnose and treat cancers at an early stage [4–6].

Long non-coding RNA five prime to X inactive specific transcript (LncRNA) refers to a type of non-coding RNA with a molecular length of more than 200 nucleotides. Recent studies showed that LncRNA may play an important role in regulating a variety of diseases [7–9]. Some long-chain non-coding RNAs were found that increased significantly in tumor tissues. These data suggest that the LncRNA may be used as a biomarker for cancer screening and a potential target for the evaluation of clinical prognosis in cancer patients [10].

LncFTX is located upstream of X inactive specific transcript (XIST), in the X-inactivation center (XIC). It produces a long non-coding RNA splicing sequence, which can significantly up-regulate the expression of XIST. LncFTX, encoded by FTX gene, is a highly conserved transcript with about 2300 nucleotides. Previous studies found that the lncFTX functions, as an oncogene, can regulate the progression of some cancers including renal cell carcinoma, hepatocellular carcinoma, and glioma [11–13]. In this regard, we conducted this systematic review and meta-analysis to comprehensively examine the relationship between the expression of lncFTX and the prognosis of cancer patients.

## Methods

Two independent investigators (JZ, WW) performed a literature search of the PubMed, Embase, Web of Science and Cochrane Library databases from inception until December 01, 2020 for eligible studies. The search terms used in each database were presented as follows:

PubMed: lncRNA or long non-coding RNA, FTX or five prime to XIST, survival, and cancers;

Embase: lncRNA or long non-coding RNA, FTX or five prime to XIST, prognosis, cancers;

Web of Science: lncRNA or long non-coding RNA, FTX or five prime to XIST, clinical outcome or survival, cancers or tumor or carcinoma or sarcoma;

Cochrane Library databases: lncRNA or long non-coding RNA, FTX or five prime to XIST, prognosis or survival, cancers or tumor or carcinoma or sarcoma.

After excluding duplicated publications, two investigators (JZ, WW) independently screened articles by reading titles and abstracts. The full text of papers that appear to be relevant were retrieved and screened against the eligibility criteria. Discrepancies between the two investigators were resolved by a discussion with a third author, a strategy that was used in our previous papers [14–16].

### Inclusion and exclusion criteria

Studies that met the following inclusion and exclusion criteria were eligible for this meta-analysis.

#### Inclusion criteria

1. Studies with the aim of assessing clinical-pathological or prognostic significance of lncFTX in human cancers.
2. Patients were categorized into two groups according to the expression of lncFTX (high versus low).
3. Studies provided relevant data that can be used for this meta-analysis.

#### Exclusion criteria

1. Survival outcome was not included.
2. The value of the cut-off point of a high expression of lncFTX was not provided.

### Data extraction

Two investigators (JZ, WW) extracted the information of eligible studies including the name of first author, year of publication, study design, country, type of sample, cancer type, study sample size, number of patients with high/low (H/L) expression of lncFTX, gender, inclusion period, follow-up time and method.

### Quality assessment of included studies

Newcastle Ottawa scale (NOS) was used for the assessment of methodological quality of included studies in this review. The full score of the scale is 9 stars. The evaluation items mainly include object selection, comparability, outcome (cohort study) or exposure (case-control). Each item has evaluation items, and each item is indicated by star, among which the comparability item can get 2 stars. NOS has been widely used in case-control studies and cohort studies. For case-control studies: 1) selection consists of four items, each with a star: case definition, representativeness, control selection, control definition; 2) compatibility: control matches the important factors, giving a star, and the research also controls other important factors by adding another star; 3) exposure includes the following three items, each with a star: assurance of exposure, same method of assessment for cases and controls, non-response rate. In the present study, NOS was used to evaluate the quality of included articles. Studies with a NOS score > 6 stars were enrolled in the present study.

### Statistical analysis

Excel 2007 was used for data extraction and management, and Stata 12.0 was used for data analysis. I^2^ statistics was used to assess the between-study heterogeneity [17]. I^2^ > 50% (or the corresponding *P* value < 0.05) represent significant statistical heterogeneity. Fixed-effects model was used to pool the data when no or low heterogeneity was found (*P* > 0.05 and I^2^ < 50%). If there was significant heterogeneity between studies, random-effects model was used. Study results for the association between the expression of lncFTX and prognosis of multiple cancers were reported as odds ratio (OR)/hazard ratio (HR) value with 95% confidence interval (CI). Additionally, a leave-one-out sensitivity analysis was conducted to identify any influential study, and the publication bias of included studies was tested by Begg’s funnel plot and Egger’s test [18].

### Prognosis of FTX in TCGA dataset

We used The Cancer Genome Atlas (TCGA) dataset (http://gepia.cancer-pku.cn/) to compare the expression of FTX between sarcoma tissues and normal tissues. Then, we used Kaplan-Meier (KM) curve to present the association between FTX expression and overall survival (OS) and disease-free survival (DFS) of cancer [19–21].

## Results

### Search results

**Fig. 1:**
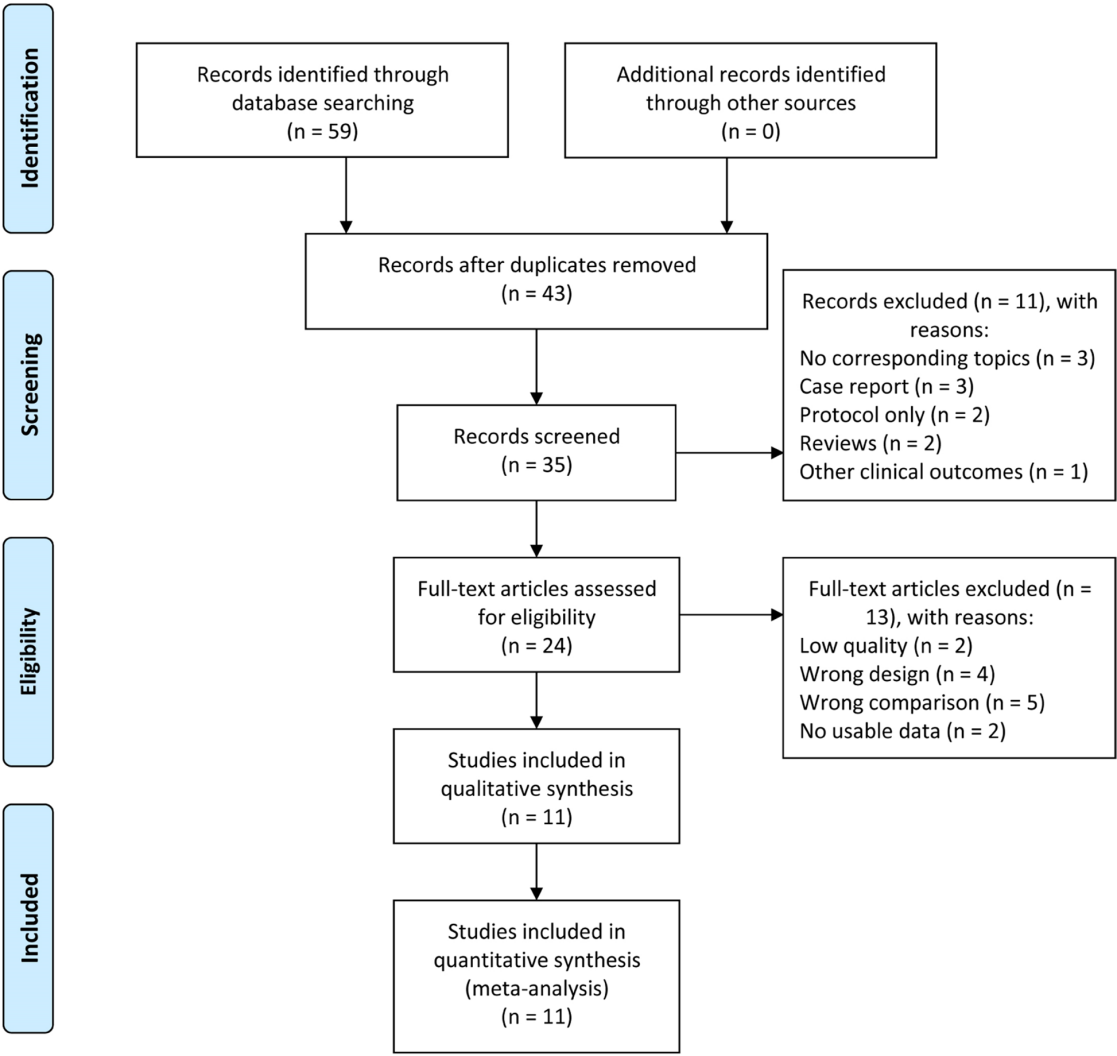
The flow figure for paper selection

We obtained 59 articles in the initial search. After removing 16 papers due to duplication and 32 papers that did not meet the eligibility criteria though the full-text reading, we included 11 papers [11–13, 22–29] published between 2015 and 2020 in the final analysis (Fig. 1).

### Characteristics of included studies and quality evaluation

A total of 11 studies with 1633 cancer patients were included in this meta-analysis. All the included studies were retrospective and most of them were conducted in China. Tissues were used for detection in these articles. The relevant malignant tumors included: glioma, colorectal cancer, gastric cancer, endometrial carcinoma, osteosarcoma, renal cell carcinoma and hepatocellular carcinoma. The sample size of these studies ranged from 30 to 543. Quantitative real-time polymerase chain reaction (qRT-PCR) was used to detect the expression of lncFTX in these included studies. These studies also provided the clinicopathological data of cancer patients, which included gender, age, tumor size, tumor differentiation, tumor node metastasis (TNM) stage, vessel invasion, lymph nodes metastasis, distant metastasis, and overall survival. For the evaluation of study quality, the NOS score of included publications ranged from 7 to 8 with an average score of 7.6 (Table 1 and Table S1).

**Table 1:** Characteristics of 11 studies for this meta-analysis

No.	First author	Year	Study design	Country	Type of sample	Tumor type	Cases	LncFTX (H/L)	Gender (M/F)	Inclusion period	Mean Follow up (month)	Method	Survival analysis	NOS score
1	Liang et al.	2020	Retrospective	China	Tissue	Glioma	187	95/92	89/98	–	60	qRT-PCR	Multivariate	8
2	Zhao et al.	2020	Retrospective	China	Tissue	Colorectal cancer	30	15/15	16/14	–	–	qRT-PCR	–	7
3	Zhang et al.	2020	Retrospective	China	Tissue	Gastric cancer	71	32/39	39/32	2012–2013	60	qRT-PCR	Univariate	8
4	Jiang et al.	2019	Retrospective	China	Tissue	Gastric cancer	93	48/45	60/33	2015–2017	–	qRT-PCR	–	7
5	Vasquez et al.	2019	Retrospective	American	Tissue	Endometrial carcinoma	543	–	–	–	60	qRT-PCR	Univariate	8
6	Li et al.	2018	Retrospective	China	Tissue	Osteosarcoma	84	39/45	49/35	2007–2010	60	qRT-PCR	Univariate	8
7	Yang et al.	2018	Retrospective	China	Tissue	Colorectal cancer	80	40/40	53/27	2014–2016	60	qRT-PCR	Univariate	8
8	He et al.	2017	Retrospective	China	Tissue	Renal cell carcinoma	160	82/78	79/81	2012–2015	–	qRT-PCR	–	7
9	Liu et al.	2016	Retrospective	China	Tissue	Hepatocellular carcinoma	126	65/61	99/27	2006–2009	60	qRT-PCR	Univariate	7
10	Liu et al.	2016	Retrospective	China	Tissue	Hepatocellular carcinoma	129	64/65	111/18	2009–2010	60	qRT-PCR	Multivariate	8
11	Guo et al.	2015	Retrospective	China	Tissue	Colorectal cancer	130	42/88	45/85	2008–2010	60	qRT-PCR	Univariate	8

### Association between the expression of lncFTX and cancer clinicopathological features

**Fig. 2:**
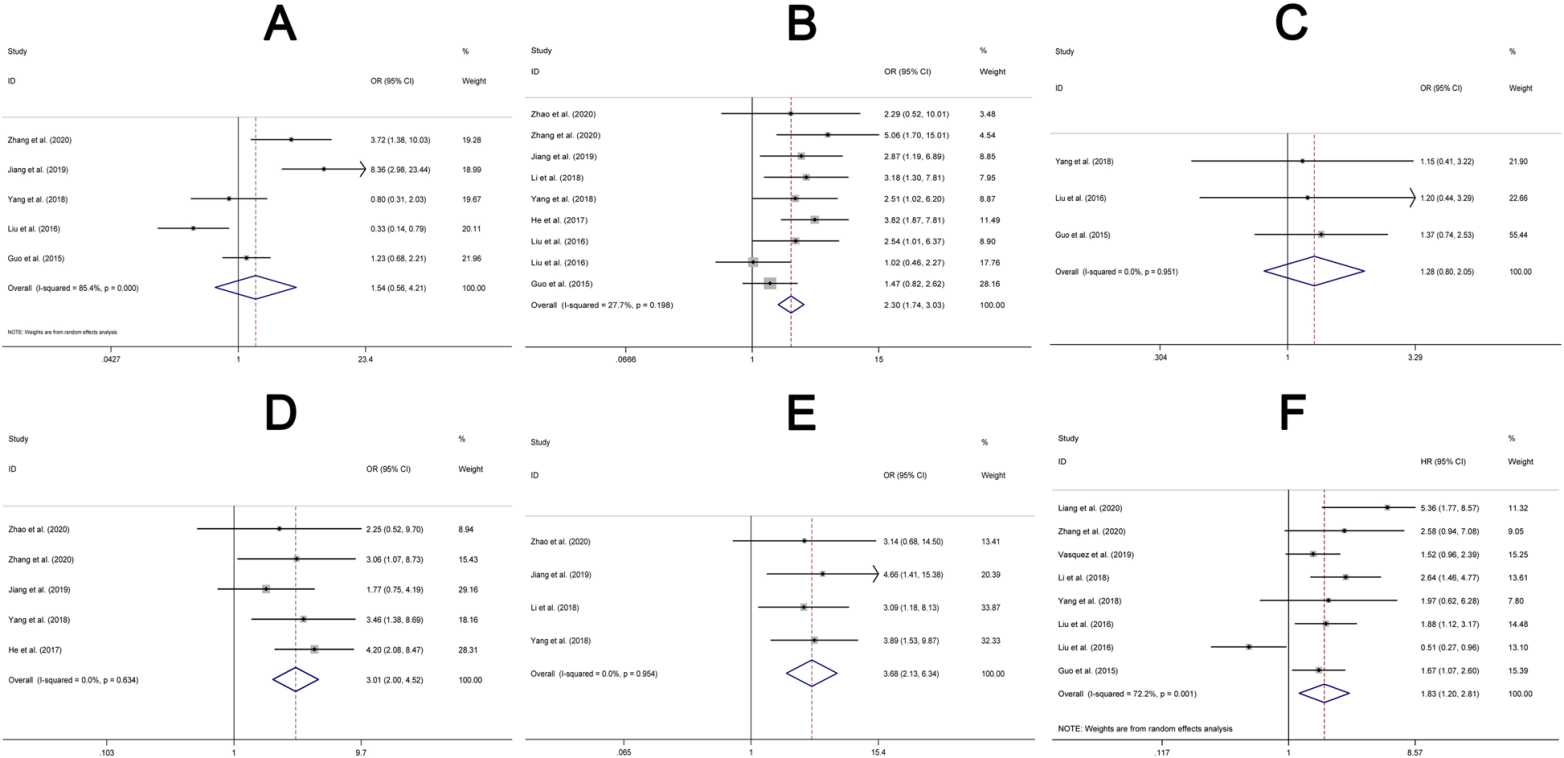
Association between lncFTX and clinical features of tumors. (**A**) tumor differentiation (PD VS WD/MD), (**B**) TNM stage (III-IV VS I-II), (**C**) vessel invasion, (**D**) lymph node metastasis, (**E**) distant metastasis and (**F**) overall survival. PD: poor differentiation, MD: moderate differentiation, WD: well differentiation

As shown in Fig. 2, we found that an elevated expression of lncFTX was significantly associated with more advanced TNM stage (III-IV VS I-II) (OR = 2.30, 95% CI: 1.74–3.03, *P* < 0.05) (Fig. 2B), greater lymph nodes metastasis (OR = 3.01, 95% CI: 2.00–4.52, P < 0.05) (Fig. 2D) and distant metastasis (OR = 3.68, 95% CI: 2.13–6.34, P < 0.05) (Fig. 2E), and shorter overall survival (HR = 1.83, 95% CI: 1.20–2.81, P < 0.05) (Fig. 2F). However, lncFTX expression was not associated with tumor differentiation (poor differentiation [PD] versus well or moderate differentiation [WD/MD]) (OR = 1.54, 95% CI: 0.56–4.21, *P* > 0.05) (Fig. 2A) or vessel invasion (OR = 1.28, 95% CI: 0.80–2.05, P > 0.05) (Fig. 2C) of cancer. OR/HR, 95% CI and the heterogeneity of this meta-analysis were presented in Table 2.

**Table 2:** Correlation between high expression of lncFTX and clinicopathologic features for tumor

Clinicopathologic features	Studies	OR/HR and 95% CI	Effects model	Heterogeneity (p; I^2^)
Tumor differentiation (PD VS Well/moderate)	5	1.54 0.56–4.21)	Random	0.000; 85.4%
TNM stage (III-IV VS I-II)	9	2.30 (1.74–3.03)	Fixed	0.198; 27.7%
Vessel invasion (Yes VS No)	3	1.28 (0.80–2.05)	Fixed	0.951; 0.0%
Lymph node metastasis (Yes VS No)	5	3.01 (2.00–4.52)	Fixed	0.634; 0.0%
Distant metastasis (Yes VS No)	4	3.68 (2.13–6.34)	Fixed	0.954; 0.0%
Overall survival	8	1.83 (1.20–2.81)	Random	0.001; 72.2%

*PD* poor differentiation, *MD* moderate differentiation, *WD* well differentiation

**Fig. 3:**
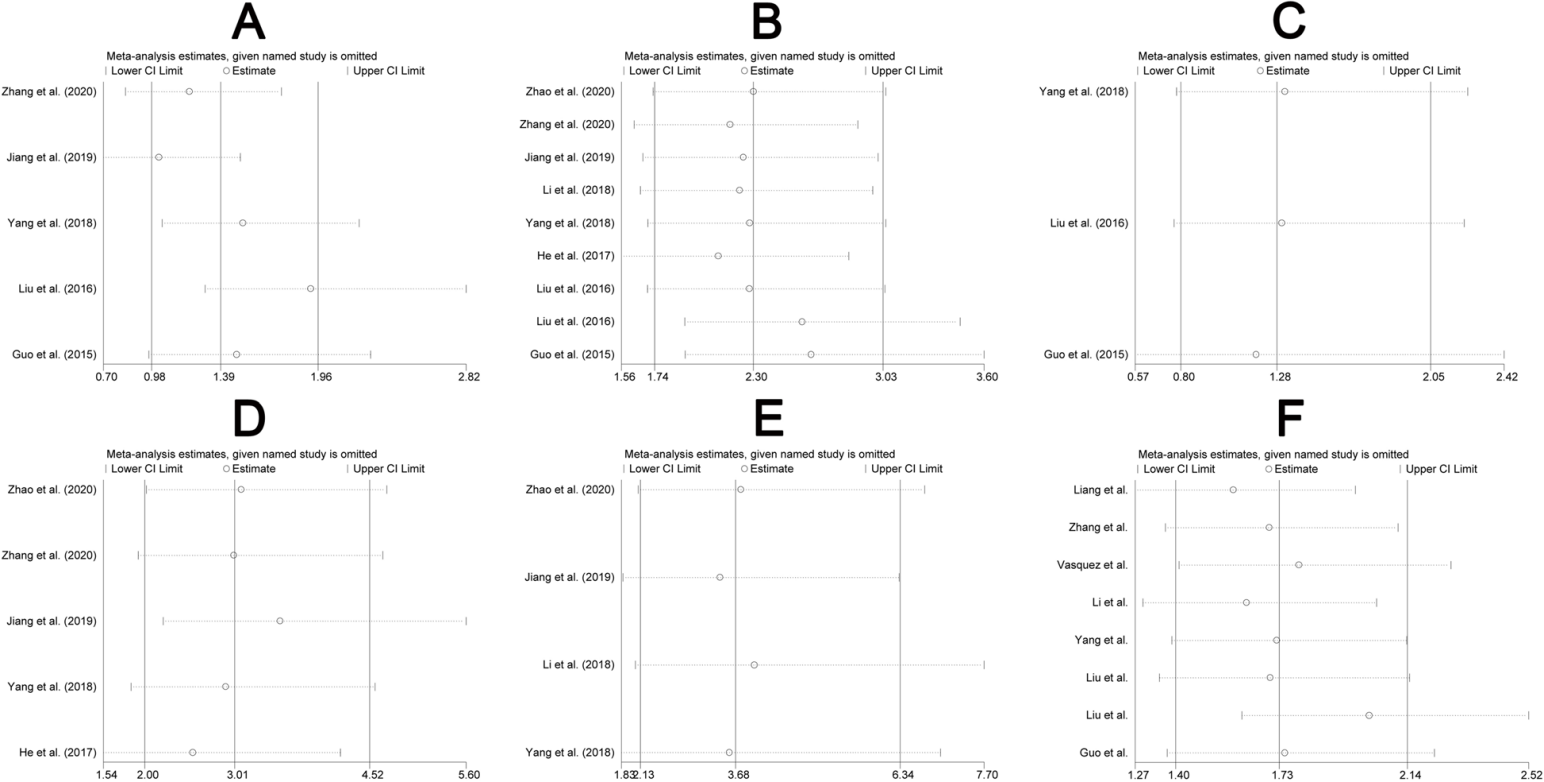
Sensitivity analysis on the relationship for lncFTX and clinical features of cancers. (**A**) tumor differentiation (PD VS WD/MD), (**B**) TNM stage (III-IV VS I-II), (**C**) vessel invasion, (**D**) lymph node metastasis, (**E**) distant metastasis and (**F**) overall survival. PD: poor differentiation, MD: moderate differentiation, WD: well differentiation

**Fig. 4:**
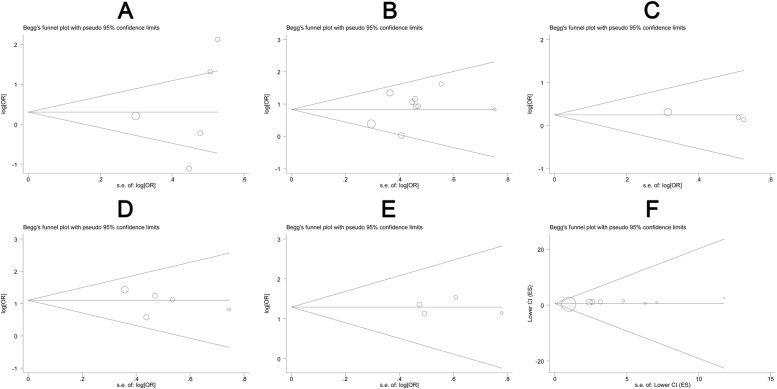
Funnel plot for association between lncFTX and clinical features of cancers. (**A**) tumor differentiation (PD VS WD/MD), (**B**) TNM stage (III-IV VS I-II), (**C**) vessel invasion, (**D**) lymph node metastasis, (**E**) distant metastasis and (**F**) overall survival. PD: poor differentiation, MD: moderate differentiation, WD: well differentiation

In sensitivity analysis by removing one study iteratively in the meta-analysis for each study outcome, the ORs with their corresponding CIs varied in the range from 1.03 (95% CI: 0.70–1.50) to 1.91 (95% CI: 1.30–2.82) for tumor differentiation (Fig. 3A), from 2.10 (95% CI: 1.56–2.84) to 2.62 (95% CI: 1.91–3.60) for TNM stage (III-IV VS I-II) (Fig. 3B), from 1.18 (95% CI: 0.57–2.42) to 1.32 (95% CI: 0.78–2.23) for vessel invasion (Fig. 3C), from 2.54 (95% CI: 1.54–4.20) to 3.52 (95% CI: 2.21–5.60) for lymph nodes metastasis (Fig. 3D), from 3.42 (95% CI: 1.85–6.33) to 3.98 (95% CI: 2.05–7.70) for distant metastasis (Fig. 3E), and the HRs varied in the range from 1.58 (95% CI: 1.27–1.97) to 2.02 (95% CI: 1.61–2.52) for overall survival (Fig. 3F). These results did not vary much as compared with the main study results, suggesting that the main study results were not driven by a single study (Fig. 3). In terms of potential publication bias, no asymmetry was observed in the funnel plot (Fig. 4) and no publication bias was found according to Egger’s test result (*P* > 0.05).

### Association between elevated expression of lncFTX and survival for cancers

**Fig. 5:**
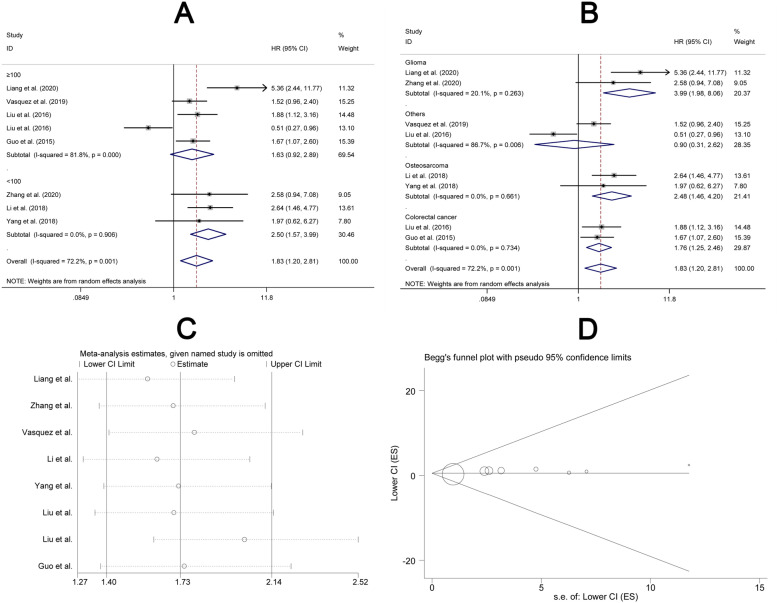
Correlation between lncFTX and survival of cancers. Subgroup analysis included (**A**) sample size, (**B**) cancer type, (**C**) sensitivity analysis, (**D**) publication bias

Results from eight included studies (Table 3) were pooled to analyze the association between high expression of lncFTX and survival rates for multiple cancers. Subgroup analyses were conducted by dividing the studies according to the sample size (≥100 and < 100) and cancer type (glioma, osteosarcoma, colorectal cancer, and others). We found that higher expression of lncFTX was associated with a greater cancer mortality in studies with sample size less than 100 (HR = 2.50, 95% CI: 1.57–3.99, *P* < 0.05) (Fig. 5A). Additionally, the results of subgroup analysis showed that elevated expression of lncFTX was associated with shortened OS of glioma (HR = 3.99, 95% CI: 1.98–8.06, P < 0.05), osteosarcoma (HR = 2.48, 95% CI: 1.46–4.20, P < 0.05) and colorectal cancer (HR = 1.76, 95% CI: 1.25–2.46, P < 0.05) (Fig. 5B). The detailed information was shown in Table 4. Considering the high heterogeneity, the data were pooled with a random effects model. Sensitivity analysis showed that the consistent result and the HR was in the range from 1.58 (95% CI: 1.27–1.97) to 2.02 (95% CI: 1.61–2.52) for overall survival (Fig. 5C). These results did not vary much as compared with the main study results, and no publication bias was found (Fig. 5D).

**Table 3:** Features of papers for the meta-analysis of 5-year survival in cancers

Trial.	Year	High expression		Low expression		Cases	Outcomes	Cancer type	Follow-up
		Death	5-year survival	Death	5-year survival				
Liang et al.	2020	86	9	64	28	> 100	OS	Glioma	60
	2020	93	2	73	19	> 100	PFS	Glioma	60
Zhang et al.	2020	26	6	20	19	< 100	OS	Glioma	60
Vasquez et al.	2019	81	190	56	216	> 100	OS	Others	60
Li et al.	2018	30	9	18	27	< 100	OS	Osteosarcoma	60
Yang et al.	2018	30	10	30	10	< 100	OS	Osteosarcoma	60
Liu et al.	2016	53	12	31	30	> 100	OS	Colorectal cancer	60
	2016	59	6	37	24	> 100	DFS	Colorectal cancer	60
Liu et al.	2016	32	32	45	20	> 100	OS	Others	60
	2016	32	32	52	13	> 100	RFS	Hepatocellular carcinoma	60
Guo et al.	2015	62	13	87	25	> 100	OS	Colorectal cancer	60
	2015	38	37	45	67	> 100	RFS	Colorectal cancer	60

*OS* over survival, *PFS* progress-free survival, *DFS* disease-free survival

**Table 4:** Subgroup analyses for the relationship between high expression of lncFTX and the survival of patients with cancer

Subgroup	Studies	HR and 95% CI	Effects model	Heterogeneity (p; I^2^)
**Cases**	8	1.83 (1.20–2.81)	Random	0.001; 72.2%
≥ 100	5	1.63 (0.92–2.89)	Random	0.000; 81.8%
< 100	3	2.50 (1.57–3.99)	Fixed	0.906; 0.0%
**Cancer types**	8	1.83 (1.20–2.81)	Random	0.001; 72.2%
Glioma	2	3.99 (1.98–8.06)	Fixed	0.263; 20.1%
Osteosarcoma	2	2.48 (1.46–4.20)	Fixed	0.661; 0.0%
Colorectal cancer	2	1.76 (1.25–2.46)	Fixed	0.734; 0.0%
Others	2	0.90 (0.31–2.62)	Random	0.006; 86.7%

### TCGA dataset analysis of prognostic value of FTX

**Fig. 6:**
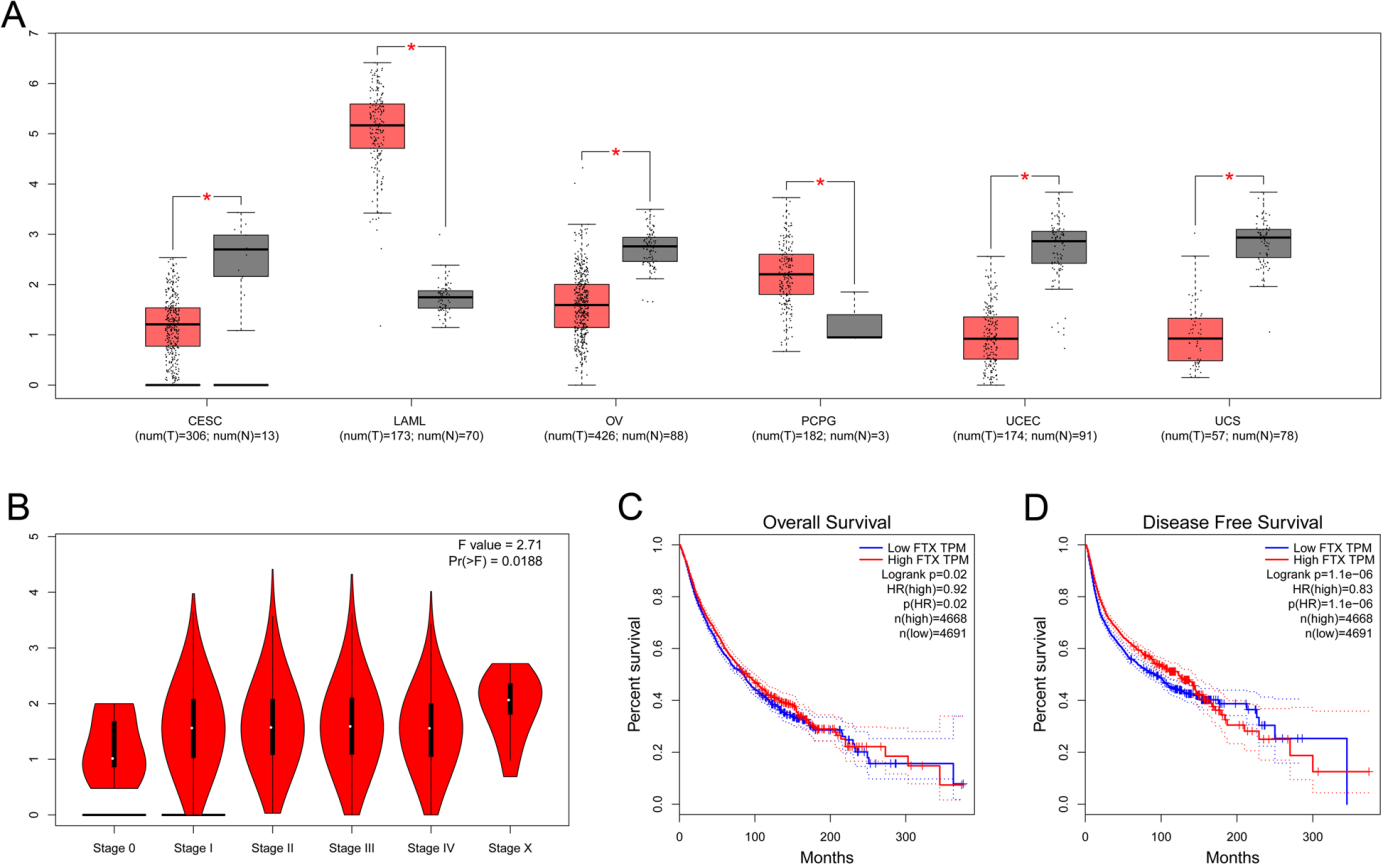
TCGA dataset analysis for the differential expression and outcome for FTX. (**A**) Differential expression of FTX between sarcoma tissue and normal tissue; (**B**) Association between FTX and clinical stage; (**C**-**D**) Correlation between FTX and (**C**) OS and (**D**) DFS in cancer

We compared the FTX expression between multiple cancers and normal tissues with using TCGA dataset. Data from 1318 cancer patients and 342 normal controls were used. We found a significant difference in the FTX expression between normal tissues and multiple cancer types including cervical squamous cell carcinoma and endocervical adenocarcinoma (CESC), acute myeloid leukemia (LAML), ovarian serous cystadenocarcinoma (OV), pheochromocytoma and paraganglioma (PCPG), uterine corpus endometrial carcinoma (UCEC) and uterine carcinosarcoma (UCS) (Fig. 6A).

Moreover, we found that the expression of FTX was significantly related to the advanced stage of cancers (*p* < 0.05) (Fig. 6B). Furthermore, we used data from 4668 patients with high FTX expression and 4691 patients with low FTX expression to analyze the prognostic role of FTX in cancer. We found that FTX was significantly associated with OS (p < 0.05) (Fig. 6C) and DFS (p < 0.05) (Fig. 6D).

## Discussion

Cancer remains a leading cause of death globally, accounting for up to 10 million deaths in 2020. In the past few decades, the association between human genes and tumor occurrence has raised growing interests. Several recent studies pointed out that the expression of lncRNA is closely related to the occurrence and development of a variety of tumors at cellular and molecular levels [7, 30, 31] The imbalanced lncRNA profile is widely involved in the occurrence and development of tumors, including tumor cell invasion, proliferation, migration, apoptosis, epithelial-mesenchymal transition (EMT) and tumor resistance. Studies have shown that LncRNA is dysregulated in the blood, urine, tumor tissue or other tissues in certain cancer patients, thus it may be used as a potential biomarker for cancer diagnosis [7, 32]

LncFTX was located in the X-inactivation center. Previous reports indicated that lncFTX was a potential predictor for glioma [22] and LncFTX could function as an oncogene that contributes to the development of colorectal cancer. Several studies reported that lncFTX was a potential target for the treatment of gastric cancer [23] and can regulate the development of osteosarcoma, renal cell carcinoma and hepatocellular carcinoma [12, 26, 28] These studies indicated that lncFTX may exert different functions in different types of tumors. However, given the small sample size of these studies, the study findings should be interpreted with caution.

To provide robust evidence, we conducted this meta-analysis of 11 studies comprising 1633 patients with tumors to systematically evaluate the prognostic value of lncFTX in various cancers. The results of the present study indicated that the elevated expression of lncFTX was significantly associated with a more advanced TNM stage (III-IV VS I-II) (OR = 2.30, 95% CI: 1.74–3.03, *P* < 0.05), greater lymph nodes metastasis (OR = 3.01, 95% CI: 2.00–4.52, P < 0.05), distant metastasis (OR = 3.68, 95% CI: 2.13–6.34, P < 0.05) and reduced OS (HR = 1.83, 95% CI: 1.20–2.81, P < 0.05), regardless of the cancer type and study sample size. Results from the sensitivity analysis were consistent with the main study findings and no significant publication bias were found.

The results of analysis using TCGA dataset revealed that FTX was differentially expressed in cancers including CESC, LAML, OV, PCPG, UCEC and UCS as compared with normal tissues. Additionally, a positive association between FTX and advanced stage of cancers was found (*p* < 0.05). Furthermore, in the analysis for the prognostic role of FTX in cancer including 4668 patients with high FTX expression and 4691 patients with low FTX expression, the results indicated that the higher expression of FTX was significantly associated with lower OS (p < 0.05) and DFS of cancer (p < 0.05), suggesting that the FTX can be used as a prognostic biomarker for cancers.

There are several limitations in the present study. First, most included studies were conducted in Asia. Whether the study results can be generalized to other regions is uncertain. Second, several HRs and 95% CIs were extracted from K-M curves, which may lead to bias. Finally, there were no sufficient studies including the outcomes of DFS and relapse-free survival (RFS).

## Conclusion

In conclusion, this meta-analysis was conducted to observe the association between high expression of lncFTX and prognosis of various cancers. The results showed that lncFTX was positively associated with advanced TNM stage, LNM and distant metastasis, and cancer mortality, suggesting that lncFTX might be a potential therapeutic target of cancers. Large-scale, well-conducted studies with high quality data are needed to confirm our study findings.

## Supplementary Information

**Additional file 1 Table S1**. Qualitative assessment of enrolled publications.

## Data Availability

The datasets used and/or analyzed during the current study are available from the corresponding author upon reasonable request.

## Abbreviations

lncFTX: Long non-coding RNA five prime to X inactive specific transcript
OR: Odds ratio
HR: Hazard ratio
95% CI: 95% confidence interval
TCGA: The Cancer Genome Atlas
PD: Poor differentiation
MD: Moderate differentiation
WD: Well differentiation
TNM: Tumor node metastasis
XIST: X inactive specific transcript
XIC: X-inactivation center
NOS: Newcastle Ottawa scale
K-M curve: Kaplan-Meier curve
OS: Overall survival
DFS: Disease-free survival
qRT-PCR: Quantitative real-time polymerase chain reaction
CESC: Cervical squamous cell carcinoma and endocervical adenocarcinoma
LAML: Acute myeloid leukemia
OV: Ovarian serous cystadenocarcinoma
PCPG: Pheochromocytoma and paraganglioma
UCES: Uterine corpus endometrial carcinoma
UCS: Uterine carcinosarcoma
EMT: Epithelial-mesenchymal transition
RFS: Relapse-free survival
