# Supplementary material for: Prognostic significance of long non-coding RNA five prime to XIST in various cancers
Source: BMC Cancer. 2022 Jan 13;22:61. doi: 10.1186/s12885-021-09161-0 (PMC8756669; doi:10.1186/s12885-021-09161-0)
Supplement: Supplementary file 1 — Additional file 1 Table S1. Qualitative assessment of enrolled publications. [file 12885_2021_9161_MOESM1_ESM.doc]

**Table S1. Qualitative assessment of included study.**

| Column | Entries | Study | | | | | | | | | | |
| --- | --- | --- | --- | --- | --- | --- | --- | --- | --- | --- | --- | --- |
|  |  | 1 | 2 | 3 | 4 | 5 | 6 | 7 | 8 | 9 | 10 | 11 |
| Section | Is the definition adequate | ☆ | ☆ | ☆ | ☆ | ☆ | ☆ | ☆ | ☆ | ☆ | ☆ | ☆ |
|  | Representativeness of the cases | ☆ | ☆ | ☆ | ☆ | ☆ | ☆ | ☆ | ☆ | ☆ | ☆ | ☆ |
|  | Selection of controls |  |  |  |  |  |  |  |  |  |  |  |
|  | Definition of controls | ☆ | ☆ | ☆ | ☆ | ☆ | ☆ | ☆ | ☆ | ☆ | ☆ | ☆ |
| Comparability | Comparability of cases and controls on the basis of the design and analysis | ☆☆ | ☆ | ☆☆ | ☆ | ☆☆ | ☆☆ | ☆☆ | ☆ | ☆ | ☆☆ | ☆☆ |
| Exposure | Ascertainment of exposure | ☆ | ☆ | ☆ | ☆ | ☆ | ☆ | ☆ | ☆ | ☆ | ☆ | ☆ |
|  | Same method of ascertainment for cases and controls | ☆ | ☆ | ☆ | ☆ | ☆ | ☆ | ☆ | ☆ | ☆ | ☆ | ☆ |
|  | Non-Response rate | ☆ | ☆ | ☆ | ☆ | ☆ | ☆ | ☆ | ☆ | ☆ | ☆ | ☆ |
| Total scores |  | 8 | 7 | 8 | 7 | 8 | 8 | 8 | 7 | 7 | 8 | 8 |

Notes: 1. Liang et al. 2020, 2. Zhao et al. 2020, 3. Zhang et al. 2020, 4. Jiang et al. 2019, 5. Vasquez et al. 2019, 6. Li et al. 2018, 7. Yang et al. 2018, 8. He et al. 2017, 9. Liu et al. 2016, 10. Liu et al. 2016, 11. Guo et al. 2015.
